# Multiple Electrophysiological Evaluation of Carpal Tunnel Syndrome

**DOI:** 10.1055/a-2644-7508

**Published:** 2025-07-22

**Authors:** XueYan Wu, Qiuju Su, Ping Ding, Jie Ji, JiaYi Zhu

**Affiliations:** 1 Department of Functional Neurology, the Affiliated Wuxi People's Hospital of Nanjing Medical University, Wuxi People's Hospital, Wuxi Medical Center, Nanjing Medical University, Wuxi, China; 2 Department of Rehabilitation Medicine, the Affiliated Wuxi People's Hospital of Nanjing Medical University, Wuxi People's Hospital, Wuxi Medical Center, Nanjing Medical University, Wuxi, China

**Keywords:** carpal tunnel syndrome, electrodiagnostic, nerve conduction studies, median nerve, sensitivity

## Abstract

**Introduction:**

To investigate a high-sensitivity electrodiagnostic (EDX) combination for diagnosing mild carpal tunnel syndrome (CTS).

**Methods:**

A total of 68 healthy controls (HCs, 136 hands) and 91 adult patients (CTSs, 162 hands) clinically diagnosed with CTS were enrolled. All patients accepted EDXs, including the sensory ganglia segment method of the median and ulnar nerves, and motor nerve conduction of the median and ulnar nerves. We examined the electrophysiological results and compared the sensitivity and specificity of various sensory nerve detection methods for the median nerve between the two groups.

**Results:**

The electrophysiological results of the CTSs were significantly different from those of HCs. All EDX techniques selected showed high specificity (>96.3%), positive predictive value (>95.2%), and large area under the curve (0.922 as the smallest) for the diagnosis of CTSs. A comparison of the median distal sensory latencies with the ulnar distal sensory latencies in fingers 2 and 4 showed a high sensitivity of 98.1%. Comparison of the nerve conduction study between the median and ulnar nerves in the same hand is the most reliable EDX technique for diagnosing very mild CTS because of its high sensitivity and specificity.

**Conclusion:**

If clinical CTS patients exhibit normal median motor distal latency or sensory nerve conduction velocity across the wrist, a comparison of median and ulnar nerve conduction through the wrist, including M-U and M-U ringdiff, is recommended.

## Introduction

Carpal tunnel syndrome (CTS) is an important cause of hand pain and impairment caused by compression of the median nerve at the wrist. 1 It is the most common perihedral nerve entrapment of the upper limb encountered worldwide. 2 The diagnosis is primarily based on clinical evaluation and is considered when patients exhibit common symptoms like numbness, tingling, nighttime paresthesia, and/or neuritic “pins-and-needles” sensation in the radial 3.5 digits. 3

Electrophysiological testing enhances clinical evaluations by assessing the reduction in nerve conduction speed through the carpal tunnel and subsequent axonal damage. These tests confer additional diagnostic certainty beyond clinical impression by providing quantitative data. 4 Nerve conduction studies (NCSs) are currently the only tool that can quantitatively reveal the deterioration in median nerve function that occurs as CTS evolves from grade 1 to grade 4, as patients in these grades are clinically indistinguishable. 5 The exact methods chosen are very important for determining early lesions of the median nerve, which will affect the sensitivity and specificity of the results.

Electrodiagnostic (EDX) testing is performed using generally accepted standardized techniques according to the American Association of Neuromuscular and Electrodiagnostic Medicine (AANEM) summary statement. 6 Each EDX technique has a different recommendation level, which is influenced by the quality and consistency of the evidence backing it. 7 The recommended EDX studies for patients suspected of having CTS include median sensory or mixed nerve conduction studies, median motor conduction studies, needle examination of the abductor pollicis brevis (APB), ulnar and/or radial motor and sensory assessments, and needle electromyography (EMG) of the limb muscles innervated by the C5 to T1 roots. 8 9

Research has shown that evaluating sensory nerve responses is more effective than relying on absolute median nerve latency for identifying median nerve issues linked to CTS. 10 11 Evaluating the median sensory latency against the sensory latencies of the radial, ulnar, and median nerves (segments external to the carpal tunnel) yielded the highest precision in verifying a clinical diagnosis. 10 11

In this study, several EDX techniques were selected and performed on participants. This study involves a direct comparison of the diagnostic value in patients with clinical CTS, with the goal of finding a combination of EDX techniques that offers high sensitivity for early CTS diagnosis.

## Materials and Methods

### Design and Ethical Considerations

A case–control study was conducted. All the participants agreed to and comprehended the detailed electrophysiological protocols. Ethical approval was obtained from the ethics committee of our institution (approval number: KY24088).

### Participants

The initial inclusion criteria for this study were to recruit 100 participants in the CTS group and 70 participants in the healthy control group (HCs). After rigorous screening, the following exclusions were made: from the CTS group, 3 participants with other nerve injuries, 4 with a history of diabetes mellitus, and 2 with systemic lupus erythematosus; from the HCs group, 1 participant with other nerve injuries and 1 who was unable to tolerate electrophysiological testing. Consequently, from January 2023 to December 2023, 68 HCs (136 hands) were recruited through our medical center, and 91 adult patients(CTSs, 162 hands) clinically diagnosed with CTS were recruited from the outpatient clinic of orthopaedics and neurology in our center. The sample size was based on the number of observed cases within the study area throughout the research period. Patients diagnosed with CTS in this study had at least one of the following primary symptoms: (1) numbness, tingling pain, or paresthesia on the radial side of the three fingers and the radial side of the ring finger; (2) nocturnal awakening due to such sensory symptoms; and (3) a positive Tinel and/or Phalen sign. Patients with previous wrist trauma or surgery, as well as those with diabetes mellitus, pregnancy, or polyneuropathy were excluded. None of the HCs showed any symptoms of neurological damage or a history of underlying conditions, such as diabetes.

### Outcome Measures

We used the Vedi Keypoint EMG evoked potential instrument imported from Denmark to conduct upper limb nerve conduction tests in the two groups. The patients were conscious during the tests, which were conducted in an undisturbed and quiet environment with an indoor temperature maintained at approximately 24°C. The nerve conduction test sites included the median and ulnar nerve. 12 The participants were placed in the supine position with their forearms supinated. The sensitivity was configured to 5 to 20 V/div, the low- and high-frequency filters were set to 20 to 2 kHz, and the sweep speed was adjusted to 2 ms/div.

#### Sensory Ganglia Segment Method of Median Nerve

We segmented the median nerve sensory conduction using the orthodromic method. The median sensory NCS were recorded from thumb (D1), index (D2), and middle finger (D3) antidromically with standard distance of 10 cm (D1) and 14 cm (D2 and D3) between stimulation and recording electrodes. The latency, sensory nerve conduction velocities (SNCV) and amplitude (SNAP) of D1-W, D2-W, D3-W were calculated. The median nerve was stimulated at the D3 and palm, with subsequent recordings taken at the palm and wrist, with a fixed distance of 8 cm, to calculate and compare the SNCV difference between D3 to the palm and palm to the wrist (P-W). We also recorded the SNCV from wrist to elbow, which was stimulated at the wrist and recorded at the elbow (W-E).

#### Comparison of the Sensory Distal Latency of the Median to the Ulnar Nerve in the Ring Finger (M-U ringdiff) 6

Stimulation occurred 14 cm proximal to the recording electrode over either the median or ulnar nerves at the wrist level. The responses were recorded using patch electrodes placed on the ring finger. M-U ringdiff was used to calculate the latency difference between the median-ring and ulnar-ring recordings.

#### Ulnar Sensory Nerve Conduction Studies

Ulnar sensory NCS was recorded from the little finger (D5), whose stimulation was 14 cm proximal to the recording electrode. Latency, SNCV, and SNAP from D5 to the wrist (D5-W) were calculated. The difference between the median sensory distal latency and ulnar sensory distal latency was determined by comparing these two measurements. 11

#### Motor Nerve Conduction Studies

Motor studies of the median and ulnar nerves were conducted by recording compound muscle action potentials (CMAP) from the APB and abductor digiti minimi muscles. The recording electrodes (G1) were positioned over the muscle bellies, whereas the reference electrodes (G2) were placed over the distal tendinous insertions. Stimulation of the median and ulnar nerves occurred at the wrist, 8 cm proximal to G1, and at or below the elbow level. Following the subtraction of the latency differences, the forearm motor nerve conduction velocities were subsequently calculated. All responses were confirmed to be supramaximal, defined as maximal when the amplitude of the CMAP ceased to increase despite further increments in stimulus intensity. Distal motor latency (DML) from onset and CMAP amplitude were measured.

#### Bias

Given the variability in detection techniques, proficiency levels, and expertise among different electrophysiologists, this study invited a single physician to perform all electrophysiological tests on the participants. The testing environment and instruments were maintained consistently throughout the study.

### Carpal Tunnel Syndrome Grading and Criteria

We used Bland's electrophysiological grading scale 13 14 ( Table 1 ). The SNCV of W-P slowed down > 10 m/s than D3-P, M-U (D2-W compare D5-W) latency delayed ≥ 0.5ms, M-U ringdiff latency delayed ≥ 0.5ms, were considered abnormal. 11

**Table 1:** Neurophysiological grading scale for carpal tunnel syndromes

Grade of CTS	EDX abnormality
1-very mild	CTS detected only in two sensitive tests (e.g., SNCV slower of W-P, M-U, M-U ringdiff)
2-mild	Median DML < 4.5 ms with the SNCV of D3-W < 40 m/s
3-moderately severe	Median DML ≥ 4.5ms and < 6.5 ms with preserved SNAP
4-severe	Median DML ≥ 4.5 ms and <6.5 ms with absent SNAP
5-very severe	Median DML ≥ 6.5 ms with CMAP ≥ 0.2 mV
6-extremely severe	Median CMAP < 0.2mV

Abbreviations: CMAP, compound muscle action potential; CTS, carpal tunnel syndrome; DML, distal motor latency; SNAP, sensory nerve conduction amplitude; SNCV, sensory nerve conduction velocities.

### Statistical Analysis

Statistical analyses were performed using STATA 11 for Windows. The chi-square test was conducted to evaluate the consistency of the NCS outcome proportions across individual digits and aggregated values. To examine intergroup differences in the NCS results, an Independent Samples *t* -test was employed. Furthermore, an analysis was undertaken to assess the concurrent criterion validity between different NCS methodologies, with the correlation strength determined using the Pearson correlation coefficient for interval data or Spearman's rank correlation for ordinal variables. Each test for detecting CTS used a receiver operating characteristic curve, and the area under the curve (AUC) with a 95% confidence interval (95% CI) was compared for each test. 15 Statistical significance was set at *p* < 0.05.

## Results

A total of 91 patients with CTS (male/female: 24/67, mean age: 51 years) and 68 HCs (male/female: 19/49, mean age: 45 years) were enrolled in this study. Of the 91 CTS patients,71 (71/91, 78.02%) had bilateral CTS and 20 (20/91, 21.98%) had unilateral CTS. The EDX results of 162 hands (right, 86; left, 76) in the CTSs and 136 hands in the HCs were recorded. In the cohort of 162 hands, 79 hands were categorized as very mild and 83 as moderately severe. The demographic characteristics and clinical manifestations of all the patients with CTS are summarized in Table 2 .

**Table 2:** Demographics and clinical characteristics of carpal tunnel syndromes

Variables	*N* = 91, hands = 162
Age, median (range) years	50.62 (23–81)
Gender	
Males	24 (26.37)
Females	67 (73.63)
Unilateral vs. bilateral	
Unilateral	20 (21.98)
Bilateral	71 (78.02)
Side	
Right	86 (53.09)
Left	76 (46.91)
Grade	
Mild	79 (48.77)
Moderately severe	83 (51.23)

### Electrophysiological Results

SNCV from the D1/D2/D3/palm to the wrist of the CTSs was significantly slower than that of the HCs. The DML of the median nerve in the CTSs group was longer than that of the HCs. Median (D2)-ulnar(D5) and median-ulnar (both recorded at ring finger) sensory latency difference of CTSs were longer than 0.5 ms, and significantly longer than that of HCs ( *p* < 0.05). SNCV from D3 to the palm in HCs was slower than that from the palm to the wrist, while it was the opposite in CTSs ( Table 3 ).

**Table 3:** Electrophysiological results in healthy controls and carpal tunnel syndromes

Parameters	HCs	CTSs	*t/Z*	*p* -Value
DML (ms)	3.44 (3.25, 3.67)	4.50 (3.94, 5.09)	−12.54	0.000 a
D1-W (m/s)	62.47 ± 6.61	43.08 ± 8.69	21.33	0.000 a
D2-W (m/s)	66 (62.65, 69.30)	48.20 (42.70, 53.40)	12.94	0.000 a
D3-W (m/s)	64.14 ± 5.76	45.99 ± 7.84	22.40	0.000 a
W-E (m/s)	73.10 (71.40, 76.15)	72.60 (71.10, 74.93)	1.42	0.16
M-U (ms)	0.27 (0.16, 0.37)	1.05 (0.79, 1.59)	−14.64	0.000 a
M-U ringdiff (ms)	0.12 (0.07, 0.29)	1.05 (0.65, 1.75)	−14.55	0.000 a
P-W (m/s)	74.1 (67.45, 79.65)	48.10 (43.20, 52.20)	14.35	0.000 a
D3-P vs P-W (m/s)	−9.15 (−15.4, −3.6)	11.05 (4.50, 15.12)	−13.37	0.000 a

Abbreviations: CTS, carpal tunnel syndrome; DML, the distal motor latency of median; D1-W/D2-W/D3-W, SNCV from D1/D2/D3 to wrist; W-E, SNCV from wrist to elbow; M-U, median (D2)-ulnar (D5) sensory latency difference; M-U ringdiff, median-ulnar (both recorded at ring finger) sensory latency difference; P-W, SNCV from palm to wrist; D3-P versus P-W: the SNCV difference between D3 to the palm and palm to the wrist (P–W);

a Significantly different when comparing CTSs and HCs, *p* < 0.05.

### Electrophysiological Diagnostic Profiles in Carpal Tunnel Syndromes

All techniques had a high specificity for the diagnosis of CTSs. The M-U and M-U ringdiff showed higher sensitivity and specificity than the other techniques. The AUC of the M-U study was 0.992 (95% CI: 0.983–1.00), which was significantly higher than that of DML ( *p* = 0.000), D1-W ( *p* < 0.05), D2-W ( *p* < 0.05), D3-W ( *p* = 0.005), and D3-PvsP-W ( *p* < 0.001). The AUC of the M-U ringdiff was 0.989 (95% CI: 0.979–0.999), which was significantly higher than that of DML ( *p* = 0.000), D1-W ( *p* < 0.05), D2-W ( *p* < 0.05), and D3-PvsP-W ( *p* = 0.0013). The electrophysiological diagnostic profiles of all the patients with CTS are summarized in Table 4 .

**Table 4:** Electrophysiological diagnostic profiles in carpal tunnel syndromes

Parameters	Sensitivity (%)	Specificity (%)	PPV (%)	NPV (%)	AUC
DML (ms)	50	100	100	62.7	0.922
D1-W (m/s)	79.6	97.1	97	80	0.963
D2-W (m/s)	69.1	100	100	73.1	0.969
D3-W (m/s)	73.5	99.3	99.2	75.8	0.971
M-U (ms)	98.1	97.1	97.5	97.8	0.992 a
M-U ringdiff (ms)	98.1	98.1	98.1	98.1	0.989 b
P-W (m/s)	62.3	98.5	98.1	68.7	0.983
D3-P vs P-W (m/s)	61.1	96.3	95.2	67.5	0.950

Abbreviations: AUC, area under the curve; NPV, negative predictive value; PPV, positive predictive value.

a AUC differs significantly when M-U is compared against these parameters: DML ( *p* = 0.000), D1-W ( *p* < 0.05), D2-W ( *p* < 0.05), D3-W ( *p* = 0.005), D3-PvsP-W ( *p* < 0.001).

b AUC shows a significant variation when M-U is compared to the subsequent parameters: DML ( *p* = 0.000), D1-W ( *p* < 0.05), D2-W ( *p* < 0.05), D3-PvsP-W ( *p* = 0.0013).

### Electrophysiological Diagnostic Profiles in Very Mild Carpal Tunnel Syndromes

As at least two sensitivity tests were used to diagnose very mild CTS, we selected four sensitivity tests and studied them in pairs. The combinations were as follows: method 1: combined D3-W and M-U; method 2: D3-W and M-U ringdiff; method 3: D3-W and D3-PvsP-W; method 4: M-U and M-U ringdiff; method 5:M-U and D3-PvsP-W; and method 6: M-U ringdiff and D3-PvsP-W. Finally, Method 4 showed higher sensitivity than the other methods, and the AUC area was significantly larger than that of any other method ( *p* = 0.000) ( Table 5 ).

**Table 5:** Electrophysiological diagnostic profiles of methods in very mild carpal tunnel syndromes

Parameters	Sensitivity (%)	Specificity (%)	PPV (%)	NPV (%)	AUC	95% CI
Method 1	50.6	100	100	77.7	0.753	0.698–0.809
Method 2	50.6	100	100	77.7	0.753	0.698–0.809
Method 3	32.9	100	100	72.0	0.665	0.612–0.717
Method 4	94.9	100	100	97.1	0.975 a	0.950–0.999
Method 5	53.2	100	100	78.6	0.766	0.710–0.821
Method 6	53.2	100	100	78.6	0.766	0.710–0.821

Abbreviations: AUC, area under the curve; CI, confidence interval; NPV, negative predictive value; PPV, positive predictive value.

a Comparing Method 1 with Methods 2 to 6 reveals a significant difference in AUC ( *p* = 0.000).

## Discussion

CTS continues to be one of the most recognized and common types of median nerve compression, accounting for approximately 90% of all entrapment neuropathies. 2 Prolonged exposure to vibrations or forceful repetitive motions is believed to be the primary cause of CTS. Additionally, certain conditions such as diabetes, pregnancy, and severe obesity may increase the risk of developing this syndrome. 16 17 According to several previous studies, early-stage CTS will disturb the patient's sleep by a pronounced sensation of hand numbness accompanied by a feeling similar to swelling, despite the absence of visible swelling. 4 Therefore, timely identification and intervention are crucial to alleviate patient discomfort and enhance prognosis. In this study, we aimed to explore a highly sensitive and specific electrophysiological diagnostic combination for CTS.

Of the 91 CTS patients included in this study, 67(73.63%) were female patients, which affirmed that CTS was more frequent in women. 18 This may be related to the fact that women undertake more handwork (such as housekeeping and textile work). In this study, 71 (71/91, 78.02%) patients had bilateral CTS, which was much more than unilateral CTS. This has also been mentioned in a previous review. 2

In this study, most electrophysiological test results were significantly different between the CTSs and HCs. In previous studies, the NCS demonstrated high validity and reliability in confirming the clinical diagnosis of CTS, with a sensitivity exceeding 85% and a specificity of 95%. 19 In this study, all EDX techniques showed high specificity (>96.3%), positive predictive value (>95.2%), and large AUC (0.922 as the smallest) in the diagnosis of CTSs. Among these tests, D1-W, D3-W, M-U, and M-U ringdiff had a higher sensitivity (>70%). In particular, for the M-U and M-U ringdiff, the sensitivity reached 98.1%. Prior research has confirmed that when comparing median nerve distal sensory latencies with those of the radial or ulnar nerves, sensory studies exhibit greater sensitivity than motor studies. 20 In this study, the DWL with 50% sensitivity was much lower than that of all sensory conduction studies. We considered that EDX techniques of sensory nerves were still the most sensitive EDX tests for diagnosing CTS, which is consistent with the results of a previous study. 21 Due to its high sensitivity and specificity, comparing the NCS of the median with that of the ulnar nerve in the same hand (M-U and M-U ringdiff) represents the most precise EDX method for diagnosing CTS. Comparing distal segment sensory NCS of the median nerve to median sensory nerve conduction through the carpal tunnel in the same limb proved ineffective for diagnosing patients with clinically suspected CTS. Although the AUC area of D3-P vs. P-W was large (0.950), the observed sensitivity was notably below the anticipated level (only 61.1%). These findings align with those of previous investigations. 7 Conversely, some studies have indicated that segmental studies of the median nerve are more sensitive than comparative studies. 21 22 Variations in outcomes might stem from the differing levels of disease severity among the study participants and distinct methodologies, including varying abnormal test cutoff points and statistical procedures.

According to Bland's electrophysiological grading scale, 13 at least two sensitive abnormal EDX tests can diagnose very mild CTS. Several sensitive EDX tests we selected from the AANEM guidelines. 6 As is well understood, employing two complementary comparison methods that concur reduces the likelihood of false positives or false negatives. 11 By pairing these sensitive techniques, we confirmed that the combination of M-U and M-U ringdiff is the best EDX test combination for CTS diagnosis. This combination had a higher sensitivity (94.9%) and negative predictive value (97.1%) than other combinations. It also showed a significantly larger AUC than the other methods ( *p* = 0.000). The diagnostic effectiveness of the M-U and M-U ringdiff in measuring comparative values in the same hand is attributed to patients being their own controls, with nerve conduction variations affecting both nerves equally.

## Conclusion

Based on the evidence of this study, EDX tests, including sensory and motor conduction studies, can be used to confirm clinical CTS. If clinical CTS patients exhibit normal median motor distal latency or SNCV from D1 to D3 to the wrist, a comparison of median and ulnar nerve conduction through the wrist is recommended, including the M-U and M-U ringdiff. Although the segmental comparison of the median nerve was more sensitive than that of the comparative studies in many studies, including the AANEM guidelines, in this study, the sensitivity was notably below the anticipated level. The scope of this study was limited to comparing sensory conduction latency between the median and ulnar nerves. In future studies, we will expand this investigation to include a comparison with the radial nerve to further enhance our understanding.
